# Maternal total energy, macronutrient and vitamin intakes during pregnancy associated with the offspring’s birth size in the Japan Environment and Children’s Study

**DOI:** 10.1017/S0007114520001397

**Published:** 2020-04-21

**Authors:** Ehab S. Eshak, Chika Okada, Sachiko Baba, Takashi Kimura, Satoyo Ikehara, Takuyo Sato, Kokoro Shirai, Hiroyasu Iso, for the Japan Environment and Children’s Study Group

**Affiliations:** 1 Public Health, Department of Social Medicine, Graduate School of Medicine, Osaka University, Osaka 565-0871, Japan; 2 Department of Public Health and Preventive Medicine, Faculty of Medicine, Minia University, Minia 61511, Egypt; 3 Bioethics and Public Policy, Department of Social Medicine, Graduate School of Medicine, Osaka University, Osaka 565-0871, Japan; 4 Department of Public Health, Hokkaido University School of Medicine, Sapporo 060-8638, Japan; 5 Department of Maternal and Child Health Research, Division of Community Health and Research, Osaka Woman’s and Children’s Hospital, Osaka 594-1101, Japan

**Keywords:** Maternal diet, Energy, Protein, Fat, Carbohydrate, Vitamins, Baby size

## Abstract

Maternal diet during pregnancy can influence fetal growth; however, the available evidence is controversial. We aimed to assess whether maternal diet of Japanese women in mid-pregnancy can affect their offspring’s birth size via collection of questionnaire and medical record data. The studied sample was a large cohort of paired mothers and their singleton offspring (*n* 78 793) from fifteen areas all over Japan who participated in the Japan Environment and Children’s Study. The mid-pregnancy intakes of total energy, macronutrients and vitamins were lower than the recommended intakes for pregnant Japanese women. Maternal total energy intake was positively associated with the offspring’s birth weight; there was a 10-g mean difference in the offspring’s birth weight of mothers in the lowest (3026 g) *v*. highest (3036 g) quartiles of energy intake. Carbohydrate intake was positively associated with the offspring’s birth length (mean difference of 0·7 cm) and inversely associated with the ponderal index (mean difference of 0·8 g/cm^3^). Offspring of mothers in the highest *v*. lowest quartiles of total dietary fibre intake were on average 9 g heavier and had 0·3 cm longer birth length and 0·2 cm longer head circumference. The highest in reference to lowest intake quartile of vitamin C was associated with 13 g and 0·7 cm mean differences in the offspring’s birth weight and length, respectively. Several other associations were evident for maternal intakes of vitamins and the offspring’s birth size. In conclusion, maternal dietary intakes of energy, dietary fibre, carbohydrate and vitamins during pregnancy were associated with the offspring’s birth size.

Optimising maternal dietary balance could be a novel appropriate approach for potential improvement of offspring’s body composition^(1,2)^. Maternal diet during pregnancy can influence fetal growth directly^(3)^ and/or indirectly through affecting maternal body weight^(4).^ Except for Japan, maternal obesity is rapidly increasing in the developed countries and is associated with increased offspring’s birth weight^(5)^. In contrary, since 1970, a decreasing trend in the BMI of Japanese women was noticed^(6)^, and meanwhile accompanied by almost a double-fold increase in the rate of low birth weight in the country since then^(7)^. In order to keep the desired lean body shape^(8)^, Japanese women usually adopt inappropriate diet^(4)^ and continue to do so even during pregnancy^(4,9,10)^. Dietary intakes of macro- and micronutrients among Japanese women prior to and during pregnancy were lower than the Japanese dietary references^(4)^.

Previous studies that showed the association between dietary macronutrient intakes during pregnancy and birth size were inconsistent in their conclusions. Energy content^(11–13)^ and macronutrient intakes during pregnancy^(4,12–14)^ were frequently reported to be not associated with the estimated fetal body weight or birth weight. Maternal carbohydrate intake, in the Singapore Growing up towards Healthy Outcomes (GUSTO) study, was positively associated with the offspring’s birth length and accordingly inversely associated with the ponderal index^(13)^. Other studies, however, showed that the fetal growth and birth size were inversely associated with carbohydrate intake and positively associated with protein intake in early pregnancy^(3,15,16)^. Intakes of total energy, protein and fat, in all trimesters of pregnancy, were positively associated with the newborn’s weight in an Indian study^(17)^.

On the other hand, dietary and supplemental vitamins are essential for the biological activity, and their status varies widely throughout pregnancy and across populations. For example, folic acid intake during pregnancy can efficiently prevent neural tube defects of the offspring^(18)^, while maternal intakes of *n*-3 long-chain PUFA, folic acid and vitamin D have shown potential effects on the offspring’s birth weight^(17–20)^.

In addition to the scanty of studies that investigated maternal diet and the offspring’s birth size among Japanese^(4)^, the available evidence has been driven from studies that included a limited number of women^(1–4,11–20)^. In light of previous literature, it is obvious that the relation of maternal macronutrient and vitamin intakes during pregnancy and the offspring’s birth size is still unclear. Therefore, this study was conducted to investigate the prospective associations of maternal consumption of macronutrients and vitamins in early pregnancy and the offspring’s birth size in a large cohort of Japanese women.

## Methodology

### Study subjects and design

The Japan Environment and Children’s Study is a large birth cohort study that was funded by the Japan Ministry of the Environment. The detailed study protocol and methodology were described previously^(21,22)^. In brief, the Japan Environment and Children’s Study included women during early pregnancy who were attending local governmental offices that issue pregnancy and child follow-up handbooks and/or attending obstetric facilities where pregnancy follow-up was conducted. Eventually, 103 099 pregnancies from fifteen Japanese communities were registered and followed up between the years 2011 and 2014. The Japan Environment and Children’s Study protocol was in full accordance with the Helsinki declaration and was reviewed and approved by the Ministry of the Environment’s Institutional Review Board on Epidemiological Studies and by the Ethics Committees of all participating institutions. All subjects have given a written informed consent to participate.

### Diet assessment

A FFQ^(23)^ was distributed during the second/third trimester to determine the usual food consumption throughout the current pregnancy, and a total of 98 037 women completed this FFQ. The final sample in this analysis consisted of 78 793 women, after excluding women with multiple gestations, stillbirths, abortions and maternal chronic diseases that might require nutritional therapy and those with missing nutritional or covariates data (see online Supplementary Fig. S1). A standard portion size was specified for each item of the FFQ, and the response options for the intake frequency ranged from almost never to ≥7 times/d for foods and from almost never to ≥10 glasses/d for beverages. The intake frequencies were multiplied by the specified portion size, and accordingly, we estimated the daily intakes of all items in the FFQ. Nutrients’ contents of each food were driven from the Japanese food composition tables 5th revised revision^(24)^, and the daily intakes of nutrients were calculated by summing the contents from all the food items after multiplying by the frequency of consumption.

### Birth size

From the newborn medical records at birth, we obtained data about birth weight (g), birth length, head circumference and chest circumference (cm). All measurements were conducted by trained and experienced staffs according to the Japanese standardised manual^(25)^. Instructions were given to repeat the measurements for unusual measurements’ values. The ponderal index was calculated from the recorded birth weight and birth length as follows: ponderal index = weight in g/(crown–heel length in cm)^3^.

### Other covariates

Mother and offspring’s characteristics were obtained from self-administered questionnaires that were distributed at first trimester, second/third trimesters, delivery and 1 month after delivery, and from health check-ups during pregnancy, medical records and obstetricians’ data. Medical records (Dr-T1, Dr-0 m and Dr-1 m) transcriptions were performed by physicians, midwives/nurses and/or research coordinators. Therefore, a large bulk of information was obtained including maternal sociodemographic characteristics, lifestyle habits, in addition to medical, obstetrics and paediatric histories.

### Statistical analysis

The analyses of the present study were based on the data set jecs-ag-20160424. Medians and interquartile ranges of the studied women’s intakes of macronutrients and vitamins during pregnancy were compared with the estimated average requirement for Japanese pregnant women in the second trimester^(26,27)^. The proportion of women with intakes less than the estimated average requirement was given for each nutrient. The maternal and offspring’s characteristics were compared among binary stratified variables of nutrients (sufficient; at least equal to the estimated average requirement *v*. insufficient; less than the estimated average requirement) using the Wilcoxon signed-rank test for continuous variables and the *χ*^2^ test for categorical ones. Because the distributions of the nutritional variables under study were skewed, the Kruskal–Wallis test and ANCOVA of the generalised linear modelling procedure for the log-transformed intakes of nutrients were used. Accordingly, we compared the univariate and multivariable-adjusted mean differences in the offspring’s birth size indices (birth weight, birth length, head and chest circumferences and ponderal index) across the quartiles of maternal intakes of total energy, energy-adjusted (by density method) macronutrients (carbohydrate, fat, protein and total dietary fibre) and vitamins (vitamins A, K, E, D, C, B_6_, B_9_ and B_12_). The geometric means of nutrients in the generalised linear modelling procedure were adjusted for maternal age (continuous), height (continuous), education (junior high/high school, professional/vocational school/junior college, university or higher), household income (<2, 2–<4, 4–<6, ≥6 million Japanese yen), prepregnancy BMI (<18·5, 18·5–<25, 25–<30 or ≥30 kg/m^2^), net weight change during pregnancy (<7, 7–12, >12 kg), parity (0, 1, ≥2), smoking habit (never, quit before and after knowing pregnancy, current), ethanol drinking habit during pregnancy (never, former and current), mother’s thyroid disease (yes or no), use of folate supplement (yes or no), offspring’s sex (dichotomous) and gestational age at delivery (continuous). The median values of each nutrient intake in each quartile were used to calculate the *P*_for trend_ across the increasing quartiles of maternal dietary intakes via the generalised linear modelling procedure. Moreover, linear regression analyses were conducted to estimate the changes in the offspring’s birth size parameters with 1-sd increment of maternal macronutrients intakes. Probability values for statistical tests were two-tailed, and *P* < 0·05 was regarded as statistically significant with at least 80 % statistical power for testing. The SAS statistical package (version 9.4, SAS) was used for the analyses.

## Results

The main characteristics of the studied 78 793 pregnant Japanese women are given in Table 1. Macronutrient and vitamin intakes of the studied women were, in general, lower than the dietary recommended intakes for pregnant Japanese women (Table 2); the proportion of participants with intakes lower than the dietary recommended intakes ranged from 22·1 % for fat intake to 92·4 % for protein intake.

**Table 1.** Main characteristics of the studied mothers (Mean values and standard deviations; numbers and percentages)

Characteristics	*n*		%
Age (years)			
Mean		31·0	
sd		5·0	
Education level			
Junior high/high school	28 540		36·2
Higher professional school/vocational school/junior college	32 767		41·6
University/graduate school	17 200		21·8
Missing	286		0·4
Smoking			
Never	46 062		58·5
Former	28 673		36·4
Current	3397		4·3
Missing	661		0·8
Alcohol drinking			
Never	26 622		33·8
Former	49 517		62·8
Current	2123		2·7
Missing	531		0·7
Parity			
0	31 219		39·6
1	29 831		37·9
≥2	15 887		20·2
Missing	1856		2·4
Household annual income (million Japanese yen)			
<2	4162		5·3
2–<4	25 494		32·4
4–<6	24 219		30·7
≥6	19 472		24·7
Missing	5446		6·9
Prepregnancy BMI (kg/m^2^)			
<18·5	12 744		16·2
18·5–<25	58 160		73·8
25–<30	6126		7·8
≥30	1763		2·2
Hyperthyroidism			
Yes	769		1·0
No	77 591		98·5
Missing	433		0·5
Hypothyroidism			
Yes	695		0·9
No	77 665		98·6
Missing	433		0·5
Folate supplement use			
None	45 747		58·1
Once/month	1821		2·3
2–3 times/month	3972		5·0
1–3 times/week	6391		8·1
4–6 times/week	4348		5·5
Once/d	15 320		19·4
≥2 times/d	983		1·2
Missing	211		0·3
Offspring’s sex of current pregnancy			
Male	40 414		51·3
Female	38 379		48·7
Gestational age of the offspring of current pregnancy (weeks)			
Mean		38·9	
sd		1·5	

**Table 2.** During pregnancy intakes of energy, macronutrients and vitamins of the studied mothers (*n* 78 793)* (Median values and interquartile ranges (IQR); numbers and percentages)

	Median	IQR	Estimated average requirement for Japanese pregnant women in the second trimester	<Estimated average requirement	
				*n*	%
Energy (kcal/d)†	1620	1311–2015	2200 (2500)	66 138	83·9
Macronutrients					
Carbohydrate (g/d)	223·8	182·6–272·4			
Carbohydrate (%E)	55·3	50·2–60·3	57·5 %E as desired goal	48 761	61·9
Fat (g/d)	53·2	39·3–71·3			
Fat (%E)	29·8	25·6–34·1	25 %E as desired goal	17 419	22·1
Protein (g/d)	54·3	42·2–70·2	45	24 026	30·5
Protein (%E)	13·5	12·2–14·8	16·5 %E as desired goal	72 802	92·4
Total dietary fibre (g/d)	9·6	7·1–12·9	≥18	72 241	91·7
Vitamins					
Vitamin A (µg/d)	404	265–631	500	49 600	62·9
Vitamin K (µg/d)	155	102–238	150‡	37 497	47·6
Vitamin E (mg/d)	5·7	4·2–7·7	6·5‡	47 655	60·5
Vitamin D (µg/d)	3·9	2·3–6·1	8·5‡	68 722	87·2
Vitamin C (mg/d)	74	48–109	95	52 497	66·6
Vitamin B_6_ (mg/d)	0·93	0·71–1·22	1·2	57 800	73·4
Folate (µg/d)	229	166–312	400	69 524	88·2
Vitamin B_12_ (µg/d)	3·5	2·2–5·4	2·3	20 550	26·1

%E, proportion of individual energy intakes using the following energy-conversion value; protein = 4 kcal/g, fat = 9 kcal/g and carbohydrate = 4 kcal/g.

* Intakes were estimated from the FFQ during the second trimester.

† To convert energy values from kcal to kJ multiply by 4·184.

‡ Express adequate intake, where estimated average requirement could not be set due to insufficient scientific evidence.

In comparison with the proportions in the insufficient intake groups, the proportions of older women were higher in the sufficient intake group of all nutrients, except for carbohydrate. The proportions of highly educated women (university or higher) were higher, while the proportions of smoking women were lower in the sufficient *v*. insufficient intake groups of all nutrients, except for energy and carbohydrate. The proportions of alcohol users were higher in the sufficient intake group when compared with the insufficient intake group of most nutrients, except for carbohydrate. The proportions of nulliparous women and women who gained <7·0 kg during the pregnancy were lower in the sufficient *v*. insufficient intake groups for all nutrients, except for carbohydrate and fat. The mean gestational age of the offspring was lower in the sufficient than that in the insufficient intake groups of dietary variables, except for carbohydrate, fat and vitamin C (Table 3).

**Table 3.** Maternal and offspring’s characteristics according to maternal intakes of energy, macronutrients and vitamins during pregnancy (Mean values and percentages)

	Maternal											
	Mean age (years)	Highly educated (%)	Smoker (%)	Alcohol user (%)	Parity (%)			Pregnancy weight gain (%)			Offspring	
					0	1	≥2	<7 kg	7–12 kg	≥12 kg	Offspring boy (%)	Gestational age (weeks)
Energy												
Sufficient (2500 kcal§)	31·4‡	20·4‡	5·0‡	3·2‡	31·7	41·0	25·4‡	14·6	49·2	34·5‡	51·1	38·82‡
Insufficient	30·9	22·1	4·2	2·6	41·1	37·3	19·2	16·3	52·4	29·3	51·3	38·89
Macronutrients												
Carbohydrate (%E)												
Sufficient (57·5 %)	30·8‡	18·8‡	4·8‡	2·3‡	39·7	37·7	20·4	16·4	51·1	30·8‡	51·2	38·87
Insufficient	31·1	23·7	4·0	2·9	39·6	38·0	20·0	15·8	52·4	29·7	51·3	38·87
Fat (%E)												
Sufficient (25 %)	31·1‡	23·2‡	4·0‡	2·7‡	39·8	37·9	19·9†	15·8	52·3	29·8‡	51·4	38·88‡
Insufficient	30·7	17·0	5·5	2·8	39·0	37·8	21·0	16·8	50·5	31·1	51·0	38·86
Protein (%E)												
Sufficient (16·5 %)	31·4‡	23·8‡	3·8‡	2·8‡	37·0	39·5	21·3‡	15·4	52·2	30·3‡	51·2	38·86‡
Insufficient	30·1	17·4	5·4	2·4	45·7	34·1	17·5	17·4	51·2	29·7	51·4	38·90
Total dietary fibre												
Sufficient (18 g)	32·2‡	23·4‡	3·2‡	3·0‡	29·6	42·2	26·2‡	16·3	50·8	31·2	50·8	38·81
Insufficient	30·9	21·7	4·4	2·7	40·5	37·5	19·6	16·0	52·0	30·0	51·3	38·88
Vitamins												
Vitamin A												
Sufficient (500 μg)	31·6‡	25·3‡	3·7‡	3·0‡	34·2	40·8	22·9‡	15·7	52·5	29·8‡	51·3	38·86‡
Insufficient	30·6	19·8	4·6	2·5	42·8	36·1	18·5	16·2	51·6	30·3	51·3	38·88
Vitamin K												
Sufficient (150 μg)	31·6‡	24·3‡	3·4‡	2·8†	36·5	39·9	21·5‡	15·9	52·6	29·5‡	51·2	38·86†
Insufficient	30·4	19·1	5·3	2·5	43·1	35·7	18·7	16·2	51·a2	30·8	51·4	38·89
Vitamin E												
Sufficient (6 mg)	31·7‡	24·7‡	3·6‡	3·0†	35·6	40·3	21·9‡	15·4	52·0	30·5‡	51·4	38·86‡
Insufficient	30·5	20·0	4·8	2·5	42·2	36·3	19·0	16·4	51·8	29·9	51·3	38·89
Vitamin D												
Sufficient (5·5 μg)	31·5‡	22·6*	3·9*	2·9	31·4	42·8	23·6‡	16·3	51·6	30·1	51·4	38·83‡
Insufficient	30·9	21·7	4·4	2·7	40·8	37·1	19·7	16·0	52·0	30·1	51·3	38·88
Vitamin C												
Sufficient (95 mg)	31·6‡	25·0‡	3·2‡	2·7	36·7	39·9	21·2‡	15·7	52·2	30·4‡	51·4	38·87
Insufficient	30·7	20·2	4·9	2·7	41·1	36·8	19·6	16·2	51·8	30·0	51·3	38·88
Vitamin B_6_												
Sufficient (1·2 mg)	31·7‡	25·0‡	3·5‡	3·0‡	34·1	41·1	22·7‡	15·4	52·1	30·5‡	51·2	38·85‡
Insufficient	30·7	20·7	4·6	2·6	41·6	36·7	19·2	16·2	51·8	30·0	51·3	38·88
Folate												
Sufficient (400 μg)	31·9‡	23·1†	3·9*	3·1*	31·4	41·6	24·9‡	16·3	51·3	30·7‡	51·3	38·83‡
Insufficient	30·9	21·7	4·4	2·6	40·7	37·4	19·5	16·0	52·0	30·0	51·3	38·88
Vitamin B_12_												
Sufficient (2·3 μg)	31·3‡	23·4‡	4·1‡	2·9‡	37·2	39·4	21·1‡	15·9	52·3	29·7‡	51·3	38·86‡
Insufficient	30·2	17·4	5·0	2·2	46·4	33·5	17·6	16·4	50·7	31·2	51·3	38·91

The Wilcoxon rank-sum test and the *χ*^2^ test were used to compare between sufficient and insufficient groups for continuous and categorical variables, respectively (*P < 0·05, † *P* < 0·001, ‡ *P* < 0·001).

§ To convert energy values from kcal to kJ multiply by 4·184.

There were significant increases in the offspring’s mean birth weight, birth length, head circumference and chest circumference and decreases in the mean ponderal index across the increasing quartiles of maternal dietary intakes of energy, total fibre and the most studied vitamins (online Supplementary Tables S1 and S2). After adjusting for maternal and offspring’s characteristics, there was 10 g increment in the birth weight of offspring born to mothers in the highest *v*. lowest quartiles of total energy intake. Women in the highest *v*. those in the lowest quartiles of dietary fibre intake gave birth to on average 9 g heavier and 0·3 cm taller babies. Maternal carbohydrate intake was positively associated with the offspring’s birth length and inversely associated with the ponderal index; fat intake was also inversely associated with the ponderal index, whereas protein intake was not associated with birth size (Table 4). One-sd increment in maternal energy intake was associated with 3·3 g increment in birth weight and 0·1 cm increment in chest circumference of the offspring. There were about 0·2 cm increments in birth length with 1-sd increment of %E from both carbohydrate and total dietary fibre, whereas the 0·2 cm decrement in birth length was observed for 1-sd increment in %E from fat (data not shown in tables). As shown in Table 5, the intakes of fat-soluble vitamins, vitamin C and folate were associated with the offspring’s birth weight. Maternal intakes of vitamins C, D, K, B_6_, B_12_ and folate were associated with their babies’ birth length; vitamins A, E and D intakes were associated with head circumference; vitamins A, C and D intakes were associated with chest circumference, whereas intakes of vitamin K were inversely associated with the ponderal index of the offspring.

**Table 4.** Associations of maternal intakes of energy and macronutrients during pregnancy with the offspring’s birth size* (Mean values with their standard errors)

	Quartiles of energy and macronutrient intakes												*P*_trend_
	Q1			Q2			Q3			Q4			
	Mean		se	Mean		se	Mean		se	Mean		se	
Energy (kcal†)	1075			1466			1800			2650			
No. of participants		19 716			19 654			19 736			19 687		
Birth weight (g)	3026		2·0	3031		2·0	3036		2·0	3036		2·0	0·004
Birth length (cm)	48·94		0·01	48·96		0·01	48·97		0·01	48·97		0·01	0·41
Head circumference (cm)	33·18		0·01	33·19		0·01	33·19		0·01	33·20		0·01	0·34
Chest circumference (cm)	31·77		0·01	31·78		0·01	31·79		0·01	31·81		0·01	0·11
Ponderal index (kg/m^3^)	25·76		0·02	25·78		0·02	25·79		0·02	25·82		0·02	0·17
Macronutrients													
Carbohydrate (%E)	45·1			52·9			57·7			64·9			
No. of participants		19 698			19 698			19 699			19 698		
Birth weight (g)	3030		2·0	3031		2·0	3037		2·0	3030		0·20	0·07
Birth length (cm)	48·93		0·01	48·95		0·01	48·96		0·01	49·00		0·01	0·002
Head circumference (cm)	33·20		0·01	33·19		0·01	33·19		0·01	33·19		0·01	0·92
Chest circumference (cm)	31·79		0·01	31·78		0·01	31·79		0·01	31·78		0·01	0·85
Ponderal index (kg/m^3^)	25·84		0·02	25·78		0·02	25·78		0·02	25·76		0·02	0·02
Fat (%E)	21·7			27·8			31·8			38·3			
No. of participants		19 698			19 697			19 700			19 698		
Birth weight (g)	3032		2·0	3035		2·0	3033		2·0	3030		0·20	0·58
Birth length (cm)	48·98		0·01	48·97		0·01	48·95		0·01	48·94		0·01	0·10
Head circumference (cm)	33·19		0·01	33·19		0·01	33·19		0·01	33·19		0·01	0·98
Chest circumference (cm)	31·78		0·01	31·79		0·01	31·79		0·01	31·80		0·01	0·73
Ponderal index (kg/m^3^)	25·75		0·02	25·79		0·02	25·80		0·02	25·82		0·02	0·05
Protein (%E)	11·1			12·9			14·1			16·2			
No. of participants		19 698			19 697			19 700			19 698		
Birth weight (g)	3028		2·0	3035		2·0	3034		2·0	3032		2·0	0·21
Birth length (cm)	48·95		0·01	48·97		0·01	48·98		0·01	48·94		0·01	0·12
Head circumference (cm)	33·18		0·01	33·19		0·01	33·20		0·01	33·20		0·01	0·55
Chest circumference (cm)	31·79		0·01	31·79		0·01	31·78		0·01	31·79		0·01	0·75
Ponderal index (kg/m^3^)	25·78		0·02	25·79		0·02	25·77		0·02	25·82		0·02	0·23
Total dietary fibre (g/1000 kcal)	3·9			5·3			6·5			8·7			
No. of participants		19 698			19 698			19 699			19 698		
Birth weight (g)	3026		2·0	3034		2·0	3034		2·0	3035		2·0	0·02
Birth length (cm)	48·89		0·01	48·97		0·01	48·97		0·01	49·01		0·01	<0·001
Head circumference (cm)	33·17		0·01	33·19		0·01	33·21		0·01	33·19		0·01	0·05
Chest circumference (cm)	31·78		0·01	31·80		0·01	31·80		0·01	31·78		0·01	0·48
Ponderal index (kg/m^3^)	25·85		0·02	25·78		0·02	25·79		0·02	25·74		0·02	0·001

* Mean values with their standard errors were adjusted for maternal age (continuous), education (junior high school/high school, higher professional school/vocational school/junior college or university/graduate school), household income (<2, 2–<4, 4–<6 or ≥6 million Japanese yen), height, prepregnancy BMI (<18·5, 18·5–<25, 25–<30 or ≥30 kg/m^2^), pregnancy weight gain (<7, 7–12 or >12 kg), parity (0, 1 or ≥2), smoking (never, stopped before or after knowing pregnancy, current) and alcohol consumption (never, former or current), mother’s thyroid disease (yes or no), use of folate supplement (yes or no), offspring sex (boy or girl) and gestational age (continuous) according to the quartiles of nutrients after log transformation.

† To convert energy values from kcal to kJ multiply by 4·184.

**Table 5.** Associations of maternal intakes of vitamins during pregnancy with the offspring’s birth size (Mean values with their standard errors)

	Quartiles of vitamin intakes												*P*_trend_
	Q1			Q2			Q3			Q4			
	Mean		se	Mean		se	Mean		se	Mean		se	
Vitamin A (μg/1000 kcal)	136			212			289			545			
No. of participants		19 698			19 698			19 699			19 698		
Birth weight (g)	3026		2·0	3034		2·0	3036		2·0	3033		2·0	0·01
Birth length (cm)	48·94		0·01	48·96		0·01	48·97		0·01	48·97		0·01	0·16
Head circumference (cm)	33·18		0·01	33·20		0·01	33·22		0·01	33·17		0·01	0·004
Chest circumference (cm)	31·76		0·01	31·80		0·01	31·81		0·01	31·78		0·01	0·02
Ponderal index (kg/m^3^)	25·77		0·02	25·81		0·02	25·80		0·02	25·77		0·02	0·29
Vitamin K (μg/1000 kcal)	49			80			112			193			
No. of participants		19 698			19 698			19 699			19 698		
Birth weight (g)	3029		2·0	3030		2·0	3034		2·0	3037		2·0	0·05
Birth length (cm)	48·89		0·01	48·94		0·01	48·99		0·01	49·02		0·01	<0·001
Head circumference (cm)	33·18		0·01	33·19		0·01	33·20		0·01	33·20		0·01	0·45
Chest circumference (cm)	31·79		0·01	31·78		0·01	31·80		0·01	31·78		0·01	0·56
Ponderal index (kg/m^3^)	25·88		0·02	25·79		0·02	25·75		0·02	25·73		0·02	<0·001
Vitamin E (mg/1000 kcal)	2·4			3·2			3·8			5·0			
No. of participants		19 694			19 702			19 698			19 699		
Birth weight (g)	3028		2·0	3033		2·0	3038		2·0	3030		2·0	0·02
Birth length (cm)	48·94		0·01	48·95		0·01	48·99		0·01	48·96		0·01	0·20
Head circumference (cm)	33·18		0·01	33·19		0·01	33·21		0·01	33·17		0·01	0·02
Chest circumference (cm)	31·78		0·01	31·79		0·01	31·81		0·01	31·78		0·01	0·14
Ponderal index (kg/m^3^)	25·79		0·02	25·80		0·02	25·80		0·02	25·76		0·02	0·25
Vitamin D (μg/1000 kcal)	0·9			2·0			2·9			4·9			
No. of participants		19 698			19 699			19 697			19 699		
Birth weight (g)	3029		2·0	3034		2·0	3038		2·0	3038		2·0	0·006
Birth length (cm)	48·93		0·01	48·98		0·01	48·98		0·01	48·94		0·01	0·02
Head circumference (cm)	33·17		0·01	33·21		0·01	33·19		0·01	33·19		0·01	0·05
Chest circumference (cm)	31·78		0·01	31·79		0·01	31·82		0·01	31·77		0·01	0·007
Ponderal index (kg/m^3^)	25·80		0·02	25·78		0·02	25·80		0·02	25·78		0·02	0·74
Vitamin C (mg/1000 kcal)	23			38			53			85			
No. of participants		19 698			19 699			19 697			19 699		
Birth weight (g)	3023		2·0	3032		2·0	3038		2·0	3036		2·0	<0·001
Birth length (cm)	48·91		0·01	48·97		0·01	48·98		0·01	48·98		0·01	<0·001
Head circumference (cm)	33·18		0·01	33·20		0·01	33·19		0·01	33·19		0·01	0·55
Chest circumference (cm)	31·78		0·01	31·79		0·01	31·81		0·01	31·78		0·01	0·02
Ponderal index (kg/m^3^)	25·80		0·02	25·77		0·02	25·80		0·02	25·79		0·02	0·58
Vitamin B_6_ (mg/1000 kcal)	0·4			0·5			0·6			0·8			
No. of participants		19 698			19 699			19 698			19 698		
Birth weight (g)	3029		2·0	3034		2·0	3035		2·0	3032		2·0	0·23
Birth length (cm)	48·93		0·01	48·98		0·01	48·97		0·01	48·96		0·01	0·02
Head circumference (cm)	33·20		0·01	33·19		0·01	33·20		0·01	33·18		0·01	0·55
Chest circumference (cm)	31·79		0·01	31·80		0·01	31·80		0·01	31·77		0·01	0·33
Ponderal index (kg/m^3^)	25·82		0·02	25·77		0·02	25·79		0·02	25·78		0·02	0·30
Folate (μg/1000 kcal)	93			126			154			217			
No. of participants		19 698			19 698			19 697			19 700		
Birth weight (g)	3027		2·0	3035		2·0	3036		2·0	3032		2·0	0·03
Birth length (cm)	48·91		0·01	48·97		0·01	48·98		0·01	48·97		0·01	<0·001
Head circumference (cm)	33·19		0·01	33·20		0·01	33·19		0·01	33·18		0·01	0·47
Chest circumference (cm)	31·79		0·01	31·80		0·01	31·78		0·01	31·78		0·01	0·71
Ponderal index (kg/m^3^)	25·83		0·02	25·79		0·02	25·78		0·02	25·76		0·02	0·09
Vitamin B_12_ (μg/1000 kcal)	1·0			1·8			2·5			4·0			
No. of participants		19 699			19 696			19 700			19 698		
Birth weight (g)	3028		2·0	3033		2·0	3036		2·0	3032		2·0	0·08
Birth length (cm)	48·94		0·01	48·96		0·01	48·99		0·01	48·94		0·01	0·02
Head circumference (cm)	33·17		0·01	33·20		0·01	33·20		0·01	33·20		0·01	0·13
Chest circumference (cm)	31·78		0·01	31·79		0·01	31·80		0·01	31·78		0·01	0·43
Ponderal index (kg/m^3^)	25·78		0·02	25·79		0·02	25·77		0·02	25·82		0·02	0·22

* Mean values with their standard errors were adjusted for maternal age (continuous), education (junior high school/high school, higher professional school/vocational school/junior college or university/graduate school), household income (<2, 2–<4, 4–<6 or ≥6 million Japanese yen), height, prepregnancy BMI (<18·5, 18·5–<25, 25–<30 or ≥30 kg/m^2^), pregnancy weight gain (<7, 7–12 or >12 kg), parity (0, 1 or ≥2), smoking (never, stopped before or after knowing pregnancy, current) and alcohol consumption (never, former or current), mother’s thyroid disease (yes or no), use of folate supplement (yes or no), offspring sex (boy or girl) and gestational age (continuous) according to the quartiles of nutrients after log transformation.

## Discussion

In this large cohort of Japanese women, maternal dietary intakes of total energy, macronutrients and vitamins were associated with the offspring’s birth size. Meanwhile, these intakes were lower than the dietary recommended intakes for pregnant Japanese women.

Our findings that Japanese pregnant women tended to have dietary intakes lower than the RDI are matching results of previous studies^(4,9,10)^. Japanese women keenness to keep a thin body^(8)^ together with their strict adherence to the nutritional guidelines for weight control during pregnancy^(28)^ could be possible explanations of the findings of the current research.

Findings from the previous literature were a mix of discrepancies regarding the associations between maternal nutrition during pregnancy and the offspring’s birth size^(1–4,11–20,29–32)^. However, those studies that reported maternal dietary intakes during pregnancy to be associated with the offspring’s birth size justified their findings by two pathways: indirect effects through the changes in the maternal body weight^(2,4,13,15,16,29)^ and direct effects of maternal dietary intakes on the fetal growth and body composition^(1–3,11,12)^.

In contrary to our findings, many studies have shown no associations between the mother’s energy content intake and the newborn’s birth size^(4,11–14)^, and one study has reported an inverse association with neonatal abdominal obesity^(1)^. However, the positive association between maternal total energy intake and the offspring’s birth weight in our study is matching those reported in a few previous studies^(17,33)^. This could be justified by the fact that with the additional need for extra energy content during pregnancy for placental and fetal growths, the low mean energy intake of Japanese pregnant women (lower than RDI) could be under the threshold of energy intake that might be associated inversely with the offspring’s birth size.

High maternal intakes of carbohydrate were positively associated with the offspring’s birth length, and none of carbohydrate, fat or protein intakes was associated with the birth weight of the offspring in the multivariable-adjusted analysis of the current study. In a study of multi-ethnic Asian population, the addition of carbohydrate in non-energetic addition models and the substitution of carbohydrate for protein in energetic substitution models were positively associated with the newborn’s birth length, and there were no associations of carbohydrate, fat or protein intakes with the birth weight^(13)^. The majority of the previous studies showed no associations of both carbohydrate and fat intakes during pregnancy with birth weight^(4,11,12,14)^ and head circumference^(14)^ of the newborn. However, positive associations were also documented in some other studies^(13,17,29,30)^. In contrary, one Japanese study showed that dietary patterns during pregnancy rich in bread, soft drinks and confectioneries and low in vegetables and fish have inverse associations with the baby’s birth weight^(34)^.

The association between protein intake of mothers and the offspring’s birth size lost its significance in the multivariable-adjusted model. Some of the released investigations reported positive associations of the mother’s protein intake with birth weight^(2,15,17,29,33)^, inverse associations with birth weight^(16)^ and birth length^(13)^, and no association^(4,12–14)^ with birth weight of the offspring.

Optimal dietary fibre intake during pregnancy is important for both the mother’s health and fetal growth^(35)^. Higher intakes of insoluble dietary fibre by pregnant Chinese women were positively associated with their offspring’s birth weight^(35)^. Our study is the first, up to our knowledge, to investigate and to show positive associations between maternal intakes of dietary fibre and the offspring’s birth weight, birth length, head circumference and inverse associations with the ponderal index.

The positive associations between maternal dietary intakes of most studied vitamins with the offspring’s birth size in our study are matching the findings from some previous reports. Maternal intakes of vitamin D and folic acid were positively associated with the birth weight^(17–19,31,36)^. Vitamin C intake in early pregnancy was the only nutrient to show a positive association with birth weight in prospective studies in south England^(12)^ and China^(36)^. Folate dietary intakes, measured at several occasions during pregnancy, were positively associated with the birth weight in an Indian study^(17)^, while folate supplementation during pregnancy was positively associated with birth weight but not birth length or head circumference, especially in women with high BMI in low-income countries^(20)^. On the other hand, a German study showed that the mother’s intake of vitamins was not associated with their newborn’s birth weight^(33)^. To the contrary, a study in New Zealand has shown that higher intakes of vitamin B during pregnancy were inversely associated with the birth size of winter born offspring^(30)^ and a Chinese study reported that folate intake during pregnancy was inversely associated with the offspring’s birth weight^(36)^.

The huge discrepancies in the results of the studies that composed the previous literature on the one hand, and between findings of our study and those from the previous studies on the other hand can be attributed to the large differences in the population culture, characteristics and dietary habits, sample sizes, adjustment for maternal and offspring characteristics and tools used for dietary assessment. Therefore, with the large sample size of our study, the use of a validated FFQ that covered almost all the common and uncommonly consumed foods and beverages, and the adjustment of maternal and offspring characteristics, we believe that the generalisability of our study findings to the Japanese population is plausible. Yet, with the unique low dietary intakes of pregnant Japanese women, even before pregnancy, the generalisability of the findings to other populations cannot be guaranteed. Meanwhile, despite the significant differences in the outcome variables across the nutrients’ categories, the absolute values of these differences are minimal and might lack clinical meaning in the neonatal field. However, such small significant changes in the birth size parameters were also reported in the previous studies^(10,13,17)^. Moreover, the proportions of offspring with low birth weight, <2500 g across the quartiles of maternal total energy intake in our study, were 8·4 % in Q1, 7·6 % in Q2, 7·2 % in Q3 and 7·1 % in Q4; *P*_for trend_ < 0·001. Other limitations of the current study include the possibility of multicollinearity of the maternal intakes of nutrients because of common dietary sources. Thus, the contribution of other unstudied maternal dietary factors towards the offspring’s birth size cannot be excluded. The observed associations depended mainly on early- to mid-pregnancy dietary intakes that were measured once, at the time of the second/third trimester questionnaire. Dietary intakes could have changed throughout pregnancy; however, some studies have not only shown that measuring the maternal diet in early pregnancy is important for various organs’ differentiation and development^(3,12,18–20)^, but also indicated that dietary intakes are likely to be relatively constant throughout pregnancy^(37,38)^. Moreover, the same FFQ was distributed to the same cohort of women in our study in the first trimester asking about the previous 1-year intakes and showed similar low dietary intakes than the Japanese dietary recommended intakes.

### Conclusion

In summary, dietary intakes of energy, macronutrients and vitamins among Japanese pregnant women were associated with the offspring’s birth size. The offspring’s birth weight was related mainly to maternal intakes of total energy, dietary fibre and vitamins A, K, E, D, C and folate, whereas the birth length was related to maternal carbohydrates, total fibre, vitamins K, C, D, B_6_, B_12_ and folate intakes. Accordingly, the ponderal index, a proxy for the offspring’s overall adiposity, was inversely associated with maternal carbohydrates, total fibre and vitamin K intakes. Head circumference was positively associated with vitamins A, E and D intakes, while chest circumference was associated mainly with the mother’s intake of vitamins A, D and C. These findings could help postulate the pregnancy dietary guidelines to prevent unfavourable birth size of the Japanese women’s offspring.

## Supplementary material

For supplementary material accompanying this paper visit https://doi.org/10.1017/S0007114520001397. click here to view supplementary material
