# Supplementary material for: Maternal total energy, macronutrient and vitamin intakes during pregnancy associated with the offspring’s birth size in the Japan Environment and Children’s Study
Source: Br J Nutr. 2020 Apr 21;124(6):558–66. doi: 10.1017/S0007114520001397 (PMC7525098; doi:10.1017/S0007114520001397)
Supplement: Supplementary file 1 [file S0007114520001397sup001.pdf]

Supplemental Figure I. Participants' flow chart.

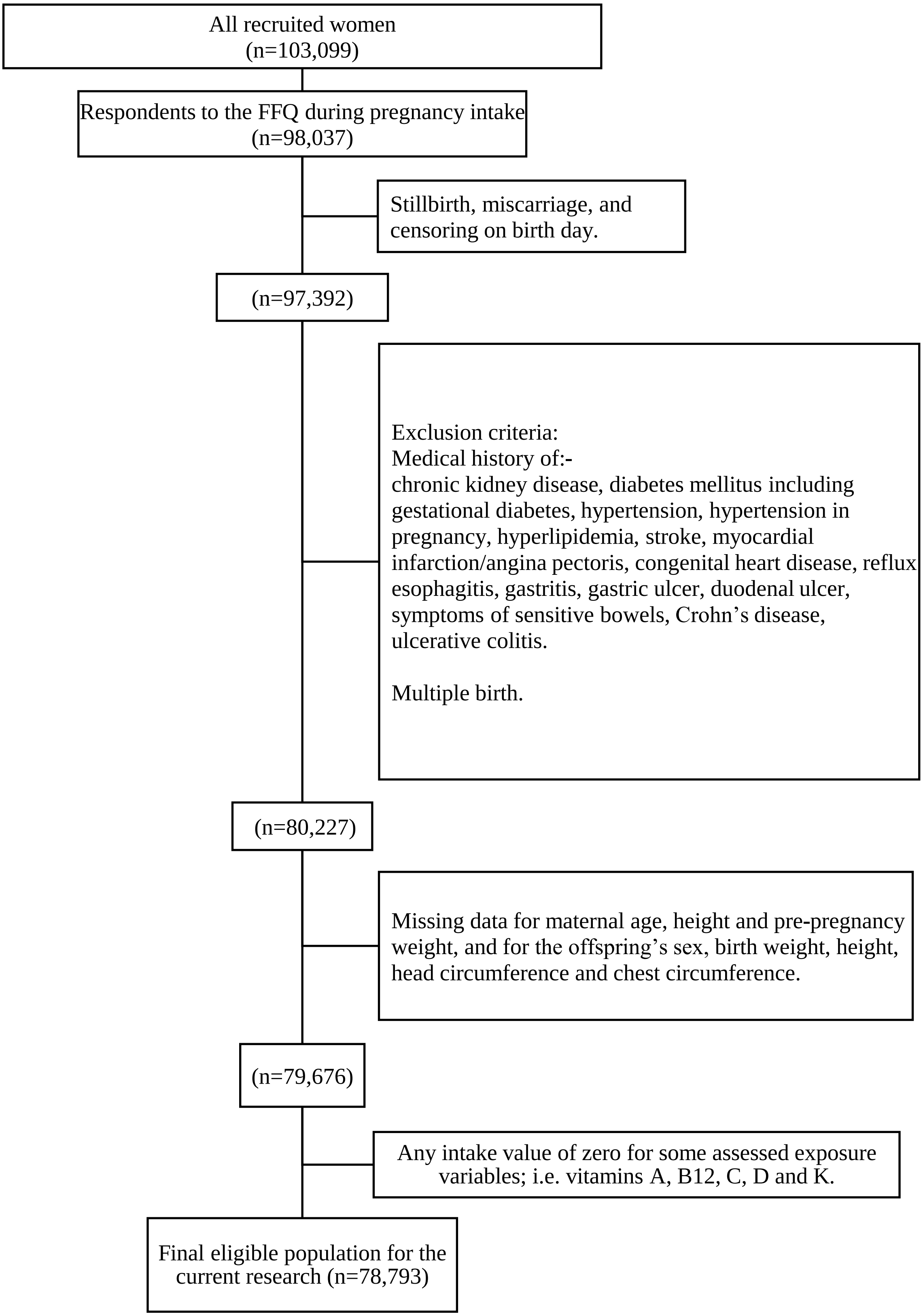

Supplemental Table I. Univariate associations of maternal intakes of total energy and macronutrients during pregnancy with offspring birth size

	Quartiles of energy and macronutrients intakes				P-value[1]
	Q1	Q2	Q3	Q4	
**Energy, median, kcal**	1,119	1,468	1,792	2,389	
Birth weight, mean (SE), g	3,013 (3)	3,029 (3)	3,041 (3)	3,046 (3)	<0.001
Birth length, mean (SE), cm	48.89 (0.02)	48.96 (0.02)	49.00 (0.02)	48.98 (0.02)	<0.001
Head circumference, mean (SE), cm	33.13 (0.01)	33.19 (0.01)	33.21 (0.01)	33.24 (0.01)	<0.001
Chest circumference, mean (SE), cm	31.72 (0.01)	31.77 (0.01)	31.81 (0.01)	31.85 (0.01)	<0.001
Ponderal index, mean (SE), kg/m³	25.72 (0.02)	25.76 (0.02)	25.80 (0.02)	25.88 (0.02)	<0.001
**Carbohydrate, median, E%**	46.4	52.9	57.6	63.8	
Birth weight, mean (SE), g	3,027 (3)	3,032 (3)	3,039 (3)	3,031 (3)	<0.001
Birth length, mean (SE), cm	48.92 (0.02)	48.96 (0.02)	49.01 (0.02)	48.95 (0.02)	<0.001
Head circumference, mean (SE), cm	33.19 (0.01)	33.20 (0.01)	33.20 (0.01)	33.18 (0.01)	0.61
Chest circumference, mean (SE), cm	31.78 (0.01)	31.79 (0.01)	31.80 (0.01)	31.78 (0.01)	0.21
Ponderal index, mean (SE), kg/m³	25.83 (0.02)	25.78 (0.02)	25.78 (0.02)	25.77 (0.02)	0.19
**Fat, median, E%**	22.6	27.8	31.8	37.3	
Birth weight, mean (SE), g	3,032 (3)	3,035 (3)	3,034 (3)	3,028 (3)	0.10
Birth length, mean (SE), cm	48.97 (0.02)	48.97 (0.02)	48.96 (0.02)	48.93 (0.02)	0.02
Head circumference, mean (SE), cm	33.18 (0.01)	33.20 (0.01)	33.20 (0.01)	33.18 (0.01)	0.55
Chest circumference, mean (SE), cm	31.78 (0.01)	31.80 (0.01)	31.79 (0.01)	31.79 (0.01)	0.71
Ponderal index, mean (SE), kg/m³	25.76 (0.02)	25.79 (0.02)	25.80 (0.02)	25.81 (0.02)	0.48
**Protein, median, E%**	11.4	12.9	14.1	15.8	
Birth weight, mean (SE), g	3,029 (3)	3,036 (3)	3,037 (3)	3,027 (3)	0.004
Birth length, mean (SE), cm	48.94 (0.02)	48.98 (0.02)	49.00 (0.02)	48.92 (0.02)	<0.001
Head circumference, mean (SE), cm	33.16 (0.01)	33.19 (0.01)	33.22 (0.01)	33.20 (0.01)	<0.001
Chest circumference, mean (SE), cm	31.78 (0.01)	31.80 (0.01)	31.79 (0.01)	31.78 (0.01)	0.21
Ponderal index, mean (SE), kg/m³	25.79 (0.02)	25.79 (0.02)	25.76 (0.02)	25.80 (0.02)	0.56
**Total dietary fiber, median, g/1000kcal**	4.1	5.4	6.4	8.2	
Birth weight, mean (SE), g	3,016 (3)	3,028 (3)	3,041 (3)	3,043 (3)	0.01
Birth length, mean (SE), cm	48.87 (0.02)	48.96 (0.02)	49.00 (0.02)	49.01 (0.02)	<0.001
Head circumference, mean (SE), cm	33.12 (0.01)	33.18 (0.01)	33.23 (0.01)	33.23 (0.01)	<0.001
Chest circumference, mean (SE), cm	31.73 (0.01)	31.78 (0.01)	31.82 (0.01)	31.83 (0.01)	0.07
Ponderal index, mean (SE), kg/m³	25.80 (0.02)	25.75 (0.02)	25.79 (0.02)	25.81 (0.02)	<0.001

SE: standard error.

[1] Kruskal-Wallis test was used assuming skewed nutritional data.

Supplemental Table II. Univariate associations beteen maternal intakes of vitamins during pregnancy and offspring birth size

	Quartiles of vitamins intakes				P-value [1]
	Q1	Q2	Q3	Q4	
**Vitamin A, median, µg/1000kcal**	143	212	286	462	
Birth weight, mean (SE), g	3,026 (3)	3,032 (3)	3,037 (3)	3,034 (3)	0.06
Birth length, mean (SE), cm	48.92 (0.02)	48.95 (0.02)	48.98 (0.02)	48.98 (0.02)	0.11
Head circumference, mean (SE), cm	33.15 (0.01)	33.19 (0.01)	33.23 (0.01)	33.19 (0.01)	<0.001
Chest circumference, mean (SE), cm	31.75 (0.01)	31.79 (0.01)	31.81 (0.01)	31.79 (0.01)	0.008
Ponderal index, mean (SE), kg/m³	25.79 (0.02)	25.80 (0.02)	25.79 (0.02)	25.77 (0.02)	0.81
**Vitamin K, median, µg/1000kcal**	51	80	110	172	
Birth weight, mean (SE), g	3,026 (3)	3,034 (3)	3,035 (3)	3,034 (3)	0.03
Birth length, mean (SE), cm	48.87 (0.02)	48.96 (0.02)	49.00 (0.02)	49.02 (0.02)	<0.001
Head circumference, mean (SE), cm	33.16 (0.01)	33.19 (0.01)	33.20 (0.01)	33.21 (0.01)	0.003
Chest circumference, mean (SE), cm	31.78 (0.01)	31.79 (0.01)	31.81 (0.01)	31.78 (0.01)	0.38
Ponderal index, mean (SE), kg/m³	25.89 (0.02)	25.80 (0.02)	25.75 (0.02)	25.71 (0.02)	<0.001
**Vitamin E, median, mg/1000kcal**	2.5	3.2	3.8	4.7	
Birth weight, mean (SE), g	3,028 (3)	3,039 (3)	3,038 (3)	3,023 (3)	<0.001
Birth length, mean (SE), cm	48.92 (0.02)	48.98 (0.02)	48.99 (0.02)	48.94 (0.02)	0.004
Head circumference, mean (SE), cm	33.16 (0.01)	33.20 (0.01)	33.22 (0.01)	33.18 (0.01)	<0.001
Chest circumference, mean (SE), cm	31.77 (0.01)	31.81 (0.01)	31.81 (0.01)	31.76 (0.01)	<0.001
Ponderal index, mean (SE), kg/m³	25.81 (0.02)	25.82 (0.02)	25.79 (0.02)	25.73 (0.02)	0.01
**Vitamin D, median, µg/1000kcal**	1.0	2.0	2.9	4.4	
Birth weight, mean (SE), g	3,028 (3)	3,035 (3)	3,039 (3)	3,027 (3)	0.01
Birth length, mean (SE), cm	48.93 (0.02)	48.98 (0.02)	48.99 (0.02)	48.94 (0.02)	0.05
Head circumference, mean (SE), cm	33.15 (0.01)	33.21 (0.01)	33.20 (0.01)	33.20 (0.01)	<0.001
Chest circumference, mean (SE), cm	31.77 (0.01)	31.79 (0.01)	31.82 (0.01)	31.77 (0.01)	0.004
Ponderal index, mean (SE), kg/m³	25.80 (0.02)	25.78 (0.02)	25.80 (0.02)	25.78 (0.02)	0.63
**Vitamin C, median, mg/1000kcal**	24	38	52	77	
Birth weight, mean (SE), g	3,019 (3)	3,034 (3)	3,041 (3)	3,034 (3)	<0.001
Birth length, mean (SE), cm	48.87 (0.02)	48.98 (0.02)	49.01 (0.02)	48.98 (0.02)	<0.001
Head circumference, mean (SE), cm	33.15 (0.01)	33.20 (0.01)	33.21 (0.01)	33.20 (0.01)	<0.001
Chest circumference, mean (SE), cm	31.74 (0.01)	31.80 (0.01)	31.83 (0.01)	31.78 (0.01)	<0.001
Ponderal index, mean (SE), kg/m³	25.82 (0.02)	25.77 (0.02)	25.80 (0.02)	25.76 (0.02)	0.23
**Vitamin B6, median, mg/1000kcal**	0.4	0.5	0.6	0.7	
Birth weight, mean (SE), g	3,027 (3)	3,038 (3)	3,036 (3)	3,028 (3)	0.009
Birth length, mean (SE), cm	48.90 (0.02)	49.00 (0.02)	48.98 (0.02)	48.95 (0.02)	<0.001
Head circumference, mean (SE), cm	33.17 (0.01)	33.20 (0.01)	33.21 (0.01)	33.19 (0.01)	0.02
Chest circumference, mean (SE), cm	31.77 (0.01)	31.81 (0.01)	31.80 (0.01)	31.76 (0.01)	0.006
Ponderal index, mean (SE), kg/m³	25.83 (0.02)	25.78 (0.02)	25.79 (0.02)	25.75 (0.02)	0.03
**Vitamin B9, median, µg/1000kcal**	97	126	154	202	
Birth weight, mean (SE), g	3,025 (3)	3,036 (3)	3,037 (3)	3,030 (3)	0.003
Birth length, mean (SE), cm	48.89 (0.02)	48.98 (0.02)	49.00 (0.02)	48.97 (0.02)	<0.001
Head circumference, mean (SE), cm	33.16 (0.01)	33.20 (0.01)	33.21 (0.01)	33.20 (0.01)	<0.001
Chest circumference, mean (SE), cm	31.77 (0.01)	31.80 (0.01)	31.80 (0.01)	31.79 (0.01)	0.18
Ponderal index, mean (SE), kg/m³	25.84 (0.02)	25.79 (0.02)	25.78 (0.02)	25.74 (0.02)	0.003
**Vitamin B12, median, µg/1000kcal**	1.1	1.8	2.5	3.7	
Birth weight, mean (SE), g	3,027 (3)	3,033 (3)	3,037 (3)	3,031 (3)	0.13
Birth length, mean (SE), cm	48.94 (0.02)	48.97 (0.02)	49.00 (0.02)	48.93 (0.02)	0.11
Head circumference, mean (SE), cm	33.15 (0.01)	33.20 (0.01)	33.21 (0.01)	33.21 (0.01)	<0.001
Chest circumference, mean (SE), cm	31.77 (0.01)	31.79 (0.01)	31.81 (0.01)	31.79 (0.01)	0.24
Ponderal index, mean (SE), kg/m³	25.78 (0.02)	25.78 (0.02)	25.77 (0.02)	25.83 (0.02)	0.28

SE: standard error.

[1] Kruskal-Wallis test was used assuming skewed nutritional data.
